# The Type of Breast Reconstruction May Not Influence Patient Satisfaction in the Chinese Population: A Single Institutional Experience

**DOI:** 10.1371/journal.pone.0142900

**Published:** 2015-11-12

**Authors:** Benlong Yang, Lin Li, Wenhui Yan, Jiaying Chen, Ying Chen, Zhen Hu, Guangyu Liu, Zhenzhou Shen, Zhimin Shao, Jiong Wu

**Affiliations:** 1 Department of Breast Surgery, Cancer Center/Cancer Institute, Fudan University, 270 Dong’an Road, Shanghai 200032, People’s Republic of China; 2 Department of Oncology, Shanghai Medical College, Fudan University, Shanghai, People’s Republic of China; 3 Department of Thyroid and Breast Surgery, ShenZhen People’s Hospital, The Second Clinical Medical College of Jinan University, Shenzhen, People’s Republic of China; 4 Department of Plastic Surgery, Zhujiang Hospital of Southern Medical University, Guangzhou, People’s Republic of China; Sapienza, University of Rome, School of Medicine and Psycology, ITALY

## Abstract

**Background:**

The goal of this study was to evaluate patient satisfaction with four common types of breast reconstruction performed at our institution: latissimus dorsi myocutaneous (LDM) flap reconstruction with or without implants, pedicled transverse rectus abdominis musculocutaneous (TRAM) flap reconstruction, and free deep inferior epigastric perforator (DIEP) flap reconstruction.

**Methods:**

A custom survey consisting of questions that assessed general and aesthetic satisfaction was sent to patients who had undergone breast reconstruction in the last 5 years. The clinical data and details of the surgery were also collected from the patients who returned the surveys. We compared satisfaction rates across the four breast reconstruction types and analyzed the effects of various factors on overall general and aesthetic satisfaction rates using a binary logistic regression model.

**Result:**

A total of 207 (72%) patients completed the questionnaires. Overall, significant differences in general and aesthetic satisfaction among the four procedures were not observed. A multivariate analysis revealed that the factor “complications” (p = 0.001) played a significant role in general satisfaction and that the factors “> 2 years since reconstruction” (p = 0.043) and “age > 35 years” (p = 0.05) played significant roles in overall aesthetic satisfaction.

**Conclusion:**

The present study demonstrated that the type of breast reconstruction might not influence satisfaction in Chinese patients.

## Introduction

The incidence of breast cancer has gradually increased in China over the last two decades,[1, 2] and with improved treatment outcomes, patients are seeking to improve their quality of life after treatment. Body disturbances after a mastectomy may result in psychological damage to the breast cancer patient[3, 4]; thus, the demand for breast reconstruction after tumor surgery, whether immediate or delayed, has increased in the present decade, especially in large cities such as Beijing and Shanghai. In fact, a single-center study in Shanghai reported that the reconstruction rate was 5.1% in 2005,[5] which had increased from 1.3% in 1990. Currently, breast cancer patients in China are increasingly concerned about their cosmetic outcomes after diagnosis. A survey of 108 post-mastectomy patients showed that 39.8% of patients felt that breast reconstruction would be a reasonable option if they could be given a second chance.[6] Patient satisfaction affects the surgeon’s choice of treatment without affecting the prognosis in plastic surgery, and over the last decade, a number of publications have focused on assessing patient satisfaction after breast reconstruction surgery.[7, 8] The general relevance of these published reports from different countries, most of which are based on Caucasian populations with variable experiences and expectations, may not be extrapolatable or applicable to the Chinese population. However, to date, large-scale studies have not been performed in a Chinese population, which is critical because different ethnic backgrounds may affect perceptions of satisfaction and quality of life. Fudan University Cancer Hospital is a leading cancer center in China that provides four types of reconstruction surgery for breast cancer patients: latissimus dorsi myocutaneous flap (LDM) reconstruction with or without implants, pedicled transverse rectus abdominis musculocutaneous (TRAM) flap reconstruction and free deep inferior epigastric perforator (DIEP) flap reconstruction. The purpose of the present study was to use a validated assessment tool to evaluate patient satisfaction with these four types of breast reconstruction post-mastectomy at our institution.

## Patients and Methods

### Study Population

Data for all patients who had undergone breast reconstruction between January 1, 2007, and January 1, 2012, were analyzed using the operating room resources and the electronic medical records (EMRs) at Fudan University Shanghai Cancer Center. A total of 285 women with breast reconstructions were identified. These patients were invited by letters or telephone to attend specially organized assessment clinics. The Dillman method was applied to the study population to increase the response rate, with non-responders contacted for a second time after 3 weeks and the remaining participants completing the questionnaire by telephone. Of the 285 patients, 207 (72%) patients agreed to participate in the study and complete the questionnaire.

### Questionnaire and Data Collection

The protocol of the present retrospective study was approved by the Ethics Committee of Fudan University Shanghai Cancer Center. A signed written informed consent form allowing the academic use of de-identified photographs and medical records was obtained from each patient. Patients' data has been stored in a safe place, accessible to the investigators only and each patient has been assigned a number. Questions assessing general and aesthetic satisfaction were previously developed and applied by the Michigan Breast Reconstruction Outcomes Study (Table 1).[7] Regarding quality of life, two questions evaluated the reconstruction’s effect on the participant’s sexual relationship and social life. These two questions were designed to provide ordinal responses on a three-point scale.[9] Additionally, all of the patients’ medical records were reviewed to collect information regarding diseases and surgery; the original resources included patient EMRs, inpatient hospital records and surgical operative notes. The measured factors included the reconstruction pattern (DIEP flap, pedicled TRAM flap, and latissimus dorsi flap with or without implants), the type of mastectomy, the age at the time of reconstruction, tumor staging, educational background, birthplace, complications, the effect on sexual life, the effect on social life, body mass index (BMI), the time interval between reconstruction and the survey, nipple reconstruction, smoking status, and postoperative radiotherapy and comorbidity. Complications included re-operation, re-hospitalization, or unplanned perioperative intravenous antibiotic treatment.[10]

**Table 1:** Satisfaction questions.

**General Satisfaction**
Question 1. Knowing what I know today, I would definitely choose to have breast reconstruction.
Question 2. Knowing what I know today, I would definitely choose to have the same type of reconstruction.
Question 3. Overall, I am satisfied with my reconstruction.
Question 4. I would recommend the type of reconstructive procedure that I had to a friend.
Question 5. I felt that I received sufficient information about my reconstruction options to make an informed choice of procedure.
**Aesthetic Satisfaction**
Question 6. The size and shape of my breasts are the same.
Question 7. My reconstructed breast(s) feel soft to the touch.

### Data Analysis

Patient characteristics were analyzed among the four main reconstructive techniques, or DIEP flap reconstruction, pedicled TRAM flap reconstruction, and latissimus dorsi flap reconstruction with or without implants. A chi-square analysis or Fisher’s exact test was used to compare the distributions of categorical variables among the four main reconstructive groups. To evaluate the effects on social and sexual relationships, the survey questions were designed to provide ordinal responses on a three-point scale. A score of one was defined as “decreased;” two, as “unaffected;” and three, as “improved.”

The primary outcome measures included general and aesthetic satisfaction. As shown in Table 1, five questions were designed to assess general satisfaction, and other two questions were designed to assess aesthetic satisfaction. The responses were scored using a five-point Likert scale ranging from very satisfied to very dissatisfied. The responses were dichotomized, with scores of 4 and 5 considered as “satisfied” and all other scores classified as “not satisfied.” Patient classification as having overall general satisfaction required a “satisfied” answer for each of the five related questions, and a classification as having overall aesthetic satisfaction required a “satisfied” answer for the two related questions.

A binary logistic regression model with backward stepwise elimination was used to determine the effects of various factors on the patient satisfaction rates of the reconstruction types. The following factors were evaluated as potential predictors of satisfaction: age at reconstruction, tumor staging, educational background, birthplace, complications, effect on sexual life, effect on social life, BMI, time interval from surgery to investigation, nipple reconstruction and reconstruction patterns. Continuous variables such as the age at reconstruction, BMI, and the time interval between the reconstruction and the survey were transformed into categorical variables before modeling. The data analysis was performed with SPSS (IBM; Chicago, IL, USA) software version 18.0. The statistical significance was set at p < 0.05.

## Results

The characteristics of the responders based on the different patterns of reconstruction are shown in Table 2. Overall, of the 285 patients initially involved in the study, 84 (29.5%) patients had chosen DIEP flap surgery, whereas the numbers of patients who had chosen pedicled TRAM flap reconstruction (20.7%), latissimus flap reconstruction (24.5%) or latissimus flap reconstruction with implants (25.3%) were 59, 70, and 72, respectively. The patient response rate was 72.6%, and 58.9% of patients had a college or university degree. In the group with latissimus dorsi flap reconstruction without implants, ductal carcinoma in situ was the most common diagnosis (26.5%). Patients who were 35 years of age or younger were more likely to have undergone latissimus flap reconstruction with or without implants (50.8% and 45.2%, respectively) than TRAM or DIEP flap reconstruction (13.6% and 8.1%, respectively). Nearly all (98.4%) DIEP flap reconstructions had been performed within 2 years of the survey. More than 60% of latissimus-based reconstructions had been performed on patients who resided in other provinces of China.

**Table 2:** Population characteristics of surgery responders based on reconstruction type.

	DIEP	Pedicled TRAM	LDMF	LDMF+IM	p
**Response rate**	73.80%	74.60%	70.10%	72.20%	
(Responders/total surveyed)	(62/84)	(44/59)	(49/70)	(52/72)	
**Type of mastectomy**					
Modified radical mastectomy	17 (27.4%)	19 (43.2%)	30 (61.2%)	42 (80.8%)	< 0.001
Simple mastectomy + sentinel lymph node biopsy	40 (64.5%)	25 (56.8%)	17 (34.7%)	9 (17.3%)	
Simple mastectomy	5 (8%)	0	2 (4%)	1 (1.9%)	
**Reconstruction stage**					
Immediate	57 (91.9%)	41 (93.2%)	49 (100%)	52 (100%)	0.045
Delayed	5 (8.1%)	3 (6.8%)	0	0	
**Education**					
College/university degree(s)	34 (54.8%)	20 (45.5%)	34 (69.4%)	32 (61.5%)	0.249
Less than college degree	20 (32.3%)	21 (47.7%)	15 (30.6%)	20 (38.5%)	
Unknown	8 (12.9%)	3 (6.8%)	0	0	
**Tumor staging**					
Ductal carcinoma in situ	9 (14.5%)	2 (4.5%)	13 (26.5%)	5 (9.6%)	0.038
Stage I	23 (37.1%)	21 (47.7%)	10 (20.4%)	24 (46.2%)	
Stage II	23 (37.1%)	17 (38.6%)	23 (46.9%)	19 (36.5%)	
Stage III	3 (4.8%)	3 (6.8%)	2 (4.1%)	4 (7.7%)	
Unknown	4 (6.4%)	1 (2.3%)	1 (2.0%)	0	
**Age at reconstruction**					
≤ 35 years	5 (8.1%)	6 (13.6%)	20 (40.8%)	26 (50.0%)	0.0001
> 35 years	56 (90.3%)	37 (84.1%)	29 (59.2%)	26 (50.0%)	
Unknown	1 (1.6%)	1 (2.3%)	0	0	
**Body mass index**					
Average (18–24)	55 (88.7%)	37 (84.1%)	44 (89.8%)	42 (80.8%)	0.648
Overweight (24–28)	5 (8.1%)	7 (15.9%)	4 (8.2%)	8 (15.4%)	
Obese (> 28)	2 (3.2%)	0	1 (2.0%)	1 (1.9%)	
Unknown	0	0	0	1 (1.9%)	
**Time interval between the reconstruction and the survey**					
Two years or less than 2 years	61 (98.4%)	35 (79.5%)	36 (73.5%)	42 (80.8%)	0.002
More than 2 years	1 (1.6%)	8 (18.2%)	13 (26.5%)	10 (19.2%)	
Unknown	0	1 (2.3%)	0	0	
**Birthplace**					
Shanghai	35 (56.5%)	24 (54.5%)	21 (42.9%)	19 (36.5%)	0.123
Outside state	27 (43.5%)	20 (45.5%)	28 (57.1%)	33 (63.5%)	
Unknown	0	0	0	0	

### Complications and Quality of Life

Patients who had undergone pedicled TRAM flap reconstruction were more likely to have developed complications (40.9%) than patients with any other reconstruction pattern. Meanwhile, patients who had undergone DIEP flap reconstruction were less likely to report that breast reconstruction had improved their sexual life compared with patients who had chosen another reconstruction type (1.6% versus 34.1%, 14.5% and 16.9%, pedicled TRAM, LDMF, LDMF+IM, respectively). Lastly, patients who had undergone DIEP flap reconstruction were more likely to have chosen nipple reconstruction (24.2%). (Table 3)

**Table 3:** Complications and quality of life.

Reconstruction type	DIEP	Pedicled TRAM	LDMF	LDMF+IM	P
**Complications**					
Yes	11 (17.7%)	18 (40.9%)	8 (16.3%)	7 (13.5%)	0.004
No	51 (82.3%)	26 (59.1%)	41 (83.7%)	45 (86.5%)	
Unknown	0	0	0	0	
**Effect on sexual life**					
Improved quality of sexual life	1 (1.6%)	15 (34.1%)	9 (18.4%)	10 (19.2%)	0.001
No effect	46 (74.2%)	23 (52.3%)	39 (79.6%)	38 (73.1%)	
Decreased quality of sexual life	11 (17.7%)	4 (9.1%)	1 (2%)	3 (5.8%)	
Unknown	4 (6.5%)	2 (4.5%)	0	1 (1.9%)	
**Effect on social life**					
Improved quality of social life	10 (16.1%)	6 (13.6%)	2 (4.1%)	3 (5.8%)	0.257
No effect	44 (71%)	33 (75%)	44 (89.8%)	45 (86.5%)	
Decreased quality of social life	6 (9.7%)	3 (6.8%)	3 (6.1%)	3 (5.8%)	
Unknown	2 (3.2%)	2 (4.5%)	0	1 (1.9%)	

### Analysis of the Questionnaire

An analysis of the individual questions demonstrated that women who had undergone latissimus dorsi flap reconstruction with implants were more likely to answer that they would undergo reconstruction again, and they were also more likely to say that they were satisfied overall, would recommend it to a friend, and had received adequate information preoperatively, although the results were not statistically significant. These patients achieved the highest overall general satisfaction rate (63.5%), although this was also not statistically significant. Women who had undergone the latissimus dorsi flap reconstruction without implants were also more likely to say that their reconstructed breasts were soft to the touch and symmetric, although these responses were not statistically significant. These patients achieved the highest overall aesthetic satisfaction rate (51.0%), although this was not statistically significant either. Patients who had undergone DIEP flap reconstruction were more likely to say that they would select the same reconstruction type, although this was not statistically significant. In Table 4, the numbers and percentages indicate an answer of 4 or 5 (satisfied) to each question in the four groups. Overall, there were no significant differences in either general or aesthetic satisfaction among the four procedures.

**Table 4:** Satisfaction based on reconstruction type.

	DIEP	Pedicled TRAM	LDMF	LDMF+IM	p
General satisfaction					
question 1	45 (72.6%)	36 (81.8%)	42 (85.7%)	51 (98.1%)	0.003
question 2	48 (77.4%)	29 (65.9%)	34 (69.4%)	40 (76.9%)	0.482
question 3	49 (79.0%)	38 (86.4%)	45 (91.8%)	49 (94.2%)	0.069
question 4	53 (85.5%)	34 (77.3%)	44 (89.8%)	49 (94.2%)	0.088
question 5	40 (66.7%)	30 (68.2%)	37 (75.5%)	42 (80.8%)	0.327*
Overall general satisfaction	33 (55.0%)	23 (52.3%)	28 (57.1%)	33 (63.5%)	0.709*
Aesthetic satisfaction					
question 6	32 (51.6%)	22 (50.0%)	30 (61.2%)	27 (51.9%)	0.675
question 7	45 (72.6%)	31 (70.5%)	40 (81.6%)	36 (69.2%)	0.497
Overall aesthetic satisfaction	30 (48.4%)	21 (47.7%)	25 (51.0%)	22 (42.3%)	0.844
*: two missing data points					

### Logistic Regression Analyses of the Four Reconstruction Types

Logistic regression analyses were used to compare patient overall general satisfaction. A multivariate analysis revealed that the factor “complications” played a constant, significant role in general satisfaction (p = 0.001). Table 5 illustrates the relative weights of all of the variables. Patients who had developed a complication were 3.268 times more likely to be dissatisfied with their reconstruction.

**Table 5:** Predictors of overall general satisfaction and overall aesthetic satisfaction with breast reconstruction.

Variable	Odds ratio	95% CI	p
**Predictive of overall general satisfaction**			
Complications	0.306	0.147–0.635	0.001
**Predictive of overall aesthetic satisfaction**			
> 2 years since reconstruction	0.409	0.173–0.971	0.043
Age > 35 years	2.026	1.001–4.102	0.05

We also performed a similar analysis to compare overall aesthetic satisfaction. The multivariate analysis showed that the factors “age > 35 years” and “> 2 years since reconstruction” continued to significantly affect overall aesthetic satisfaction (p = 0.05 and 0.043, respectively) (Table 5). Patients who had undergone breast reconstruction more than 2 years ago were 2.445 times more likely to experience aesthetic dissatisfaction with their surgery, and patients who were older than 35 years were 2.026 times more likely to experience aesthetic satisfaction than patients who were 35 years or younger.

## Discussion

It is generally accepted that reconstruction rates are affected by age, race, country, and economic class. One published paper from New York University’s Langone Medical Center[11] reported that Asian women (34%) showed a significantly lower rate of breast reconstruction than Hispanic women did (48%). At our center, although the number of annual breast cancer surgeries is approximately 3000, the reconstruction rate over the last five years has been less than 5%. This disparity may be the reason for the growing body of literature on breast reconstruction in the Asian population, although reports have not compared patient satisfaction across all types of current reconstructive techniques.[11–13]

The present study evaluated general and aesthetic satisfaction with breast reconstruction among 207 women. The results showed that significant differences were not observed in general or aesthetic satisfaction between four types of reconstruction. Patients reconstructed with latissimus flaps with or without implants seemed more satisfied than others did, although this result was not significant. Our results are somewhat inconsistent with those reported in the literature.[7, 8, 14–16] Yueh et al. evaluated the satisfaction scores of 116 latissimus-based reconstruction patients (90 with implants and 26 without) in relation to the scores of 236 abdominal flap patients and found that abdominal reconstructions had significantly higher satisfaction scores.[8] In our study, the number of patients who had undergone latissimus-based reconstruction with or without implants was more evenly divided; therefore, implant-related complications may have been minimized. Women who had undergone latissimus-based reconstruction without implants were significantly more likely to say that they would undergo the reconstruction again, although the responses to the remaining questions did not reach statistical significance. In our experience, most Chinese women have small- to medium-sized breasts and are thus well suited for latissimus flap reconstruction without implants.

Andrade, W. N. et al. reported that the presence of postoperative complications was a particularly important indicator of dissatisfaction with reconstruction.[16] Colakoglu, S. et al. examined the direct effect of complications on the patient’s general and aesthetic satisfaction and discovered that the development of a complication was associated with increased odds of aesthetic dissatisfaction.[15] Our results are consistent with these findings. In particular, patients who developed a complication were 3.268 times more likely to be dissatisfied with their reconstruction. The disadvantages of autologous reconstruction arise from donor-site morbidity, including a high incidence of seromas with LDM flaps, abdominal wall weakness and the potential for abdominal bulges and/or hernias with TRAM flaps. Our results may be attributable to the low complication rates associated with latissimus-based flap reconstruction (LDM flap, 16.3% and LDM flap with implants, 13.5% versus DIEP flap, 17.7% and TRAM flap, 40.9%, p = 0.004), although the learning curve may have contributed to the high complication rates of abdominal-based flaps. Compared with LDM flap surgery, which was first performed at our center in 2000, TRAM and DIEP flap surgeries were first performed here in 2007 but achieved a high volume only after 2010. We propose that higher satisfaction scores similar to those for latissimus-based reconstruction will be achieved as the surgeons’ skills in abdominal-based reconstruction evolve. Saulis, A. S. et al. reported that LDM flap patients had a shorter hospitalization period and recovery time compared with pedicled TRAM flap patients (p < 0.05).[14] Lindegren, A. et al. reported that patients with irradiated breasts were more satisfied with the size and shape of breasts with latissimus-based reconstruction than with DIEP surgery.[17] Our data support these prior results, although our thought-provoking study has raised concerns from Pusic, A. L. and Alderman, A. K. with respect to the small sample size and the quality-of-life questionnaire.[18, 19]

Traditionally, physicians have believed that young patients are better candidates for latissimus-based reconstruction and that older patients are better suited for TRAM or DIEP flap reconstruction because the latter may have more abdominal tissue. In the present study, the BMIs of the latissimus dorsi flap and abdominal-based flap patients were similar (Table 2).

Certain authors have proposed using fat grafting together with LDM flap reconstruction in an immediate setting for complete breast reconstruction with excellent aesthetic results and good patient satisfaction.[20] Nevertheless, we do not use this technology at our institution, mainly because as a Chinese cancer center, we do not have equipment to harvest fat.

Certain determinants have been reported as having potential effects on patient complications, including the reconstruction type,[21] timing,[10] age,[15] obesity,[10, 22–25] and smoking.[24, 25] In our study, patients receiving pedicled TRAM flaps were more likely to develop a complication (40.9%) than patients undergoing any other reconstructive method were, indicating that free flaps may reduce the risk of fat necrosis by providing a good blood supply to the donor site. Research from Garvey, P. B. et al. showed that fat necrosis rates were significantly different between a pedicled TRAM flap group (58.5%) and a DIEP flap group (17.7%, p < 0.001).[26]

We also found that women who were older than 35 years were more likely to be satisfied with the surgery. It is thought that younger women have greater concerns regarding their mastectomy scars and higher expectations for the reconstruction results.[27, 28] The literature has also demonstrated that patient age is associated with psychosocial maladjustment after reconstruction surgery.[29]

Furthermore, according to the logistic regression results, a high quality of social life had a positive effect on overall aesthetic satisfaction. This effect was not observed in relation to sexual life, which was partly because of the limited sample size used in this study. In addition, most breast reconstructions were immediate reconstructions; therefore, the patients had not experienced the loss of breast tissues or the effect of such loss on their sexual life before reconstruction.

It is well known that in plastic surgery, patient satisfaction changes over time. Specifically, satisfaction rates decreased with time, especially in implant-based breast reconstruction patients.[30] Colakoglu, S. et al. reported that aesthetic satisfaction appears to decrease day by day, whereas more than 7 years after reconstruction, patients show a small increase in satisfaction.[15] According to the literature, satisfaction with autologous tissue reconstruction increases as time progresses. Our results showed that patients who had undergone breast reconstruction more than 2 years prior were 2.445 times more likely to be frustrated with their appearance.

Several authors have demonstrated that the type of mastectomy and the stage of breast reconstruction may positively or negatively influence patient satisfaction.[31–33] In our study, most patients had undergone immediate reconstruction. One reason for this is that insurance coverage for breast reconstruction is just for immediate surgery. Regarding the mastectomy method, it mainly depends on the stage of the disease. We compared overall aesthetic satisfaction among patients with three different mastectomy types and found no significant difference. We routinely perform skin-sparing mastectomy in patients who are likely to undergo reconstruction surgery.

There are a number of validated self-report questionnaires used to evaluate patient satisfaction, including the RAND-36, BREAST-Q,[34, 35] and Michigan Breast Reconstruction Outcomes Study tool. Worldwide, the RAND-36 questionnaire is perhaps the most widely used health-related quality-of-life survey instrument. This survey consists of 36 items that assess eight health concepts, including physical functioning, role limitations caused by physical health problems, role limitations caused by emotional problems, social functioning, emotional well-being, energy/fatigue, pain, and general health perceptions. The BREAST-Q is a condition-specific instrument, and the reconstruction module measures satisfaction using questions related to the breasts, such as softness, size and implant quality. Finally, the Michigan Breast Reconstruction Outcomes Study tool is focused on general and aesthetic satisfaction after breast reconstruction. The present study aimed to evaluate the impact of different types of surgery on patients’ feelings, and the Michigan Breast Reconstruction Outcomes Study tool best fit this aim.

## Conclusions

Doctors continue to focus on recognizing patient needs, which helps to optimize the breast reconstruction experience. The current study used a retrospective evaluation and a well-validated face-to-face survey technique and demonstrated that patterns of reconstruction might not influence Chinese breast cancer patient satisfaction. However, our survey indicates that patients do benefit from breast reconstruction following mastectomy. Thus, larger and more comprehensive studies will be required to maximize patient satisfaction during breast reconstruction surgery.
